# Epigenetic Deregulation of the Histone Methyltransferase *KMT5B* Contributes to Malignant Transformation in Glioblastoma

**DOI:** 10.3389/fcell.2021.671838

**Published:** 2021-08-10

**Authors:** Virginia López, Juan Ramón Tejedor, Antonella Carella, María G. García, Pablo Santamarina-Ojeda, Raúl F. Pérez, Cristina Mangas, Rocío G. Urdinguio, Aitziber Aranburu, Daniel de la Nava, María D. Corte-Torres, Aurora Astudillo, Manuela Mollejo, Bárbara Meléndez, Agustín F. Fernández, Mario F. Fraga

**Affiliations:** ^1^Cancer Epigenetics and Nanomedicine Laboratory, Department of Organisms and Systems Biology, Nanomaterials and Nanotechnology Research Center (CINN-CSIC), Health Research Institute of Asturias (ISPA), Institute of Oncology of Asturias (IUOPA), Rare Diseases CIBER (CIBERER) of the Carlos III Health Institute (ISCIII), University of Oviedo, Oviedo, Spain; ^2^Biobanco del Principado de Asturias, Hospital Universitario Central de Asturias (HUCA), Oviedo, Spain; ^3^Departamento de Anatomía Patológica, Hospital Universitario Central de Asturias (HUCA), Oviedo, Spain; ^4^Departamento de Patología, Hospital Virgen de la Salud (CHT), Toledo, Spain

**Keywords:** epigenetics, glioblastoma, histone methyltransferase, histone posttranslational modification, tumor suppressor

## Abstract

Glioblastoma multiforme (GBM) is the most common and aggressive type of brain tumor in adulthood. Epigenetic mechanisms are known to play a key role in GBM although the involvement of histone methyltransferase *KMT5B* and its mark H4K20me2 has remained largely unexplored. The present study shows that DNA hypermethylation and loss of DNA hydroxymethylation is associated with *KMT5B* downregulation and genome-wide reduction of H4K20me2 levels in a set of human GBM samples and cell lines as compared with non-tumoral specimens. Ectopic overexpression of *KMT5B* induced tumor suppressor-like features *in vitro* and in a mouse tumor xenograft model, as well as changes in the expression of several glioblastoma-related genes. H4K20me2 enrichment was found immediately upstream of the promoter regions of a subset of deregulated genes, thus suggesting a possible role for *KMT5B* in GBM through the epigenetic modulation of key target cancer genes.

## Introduction

Glioblastoma multiforme (GBM) is a malignant grade IV glioma which represents the most common and aggressive primary brain tumor among adults. The first line of treatment consists of surgical resection followed by radiotherapy and/or chemotherapy with the alkylating agent temozolomide (TMZ). Nevertheless, the median overall survival after treatment is only 14 months (Weller et al., 2015). Hence there is a critical need to gain a deeper understanding of this frequently fatal and heterogeneous brain cancer, and search for new therapeutic targets.

During tumorigenesis, epigenetic alterations affect different types of cancer-related genes involved in cell cycle control, DNA repair, apoptosis, or cell signaling (Feinberg et al., 2016), among others. Moreover, they can also occur at epigenetic regulator genes, thereby triggering a chain of genome-wide massive epigenetic alterations. As examples of the latter are, on the one hand, epigenetic disruption through the downregulation of different micro RNAs that modulate histone deacetylases (HDACs) expression has been reported in several cancers, such as hepatocellular carcinoma (Yuan et al., 2011), lymphoplasmacytic lymphoma (Roccaro et al., 2010), tongue squamous cell carcinoma (Kai et al., 2014), and breast cancer (Wu et al., 2014). On the other hand, disruption of the methylation signature at the promoter of genes encoding histone deacetylases *HDAC4*, *HDAC5*, and *HDAC9* has been described in papillary thyroid carcinoma (White et al., 2016). In the context of neuroblastoma and glioma, epigenetic inactivation via hypermethylation at promoter CpG island (CpGi) of the histone methyltransferase gene *NSD1* has been defined as the mechanism responsible for the altered histone methylation landscape observed in both types of tumor (Berdasco et al., 2009). In the same vein, it has been shown that DNA methyltransferases (DNMTs) encoded by *DNMT1* and *DNMT3B* are overexpressed in glioma due to, respectively, an aberrant histone code or an aberrant methylation pattern at their promoters (Rajendran et al., 2011). Recently, our laboratory has demonstrated that the expression of the epigenetic enzyme TET3 is aberrantly downregulated in GBM through epigenetic mechanisms, leading to a genome-wide reduction of 5-hydroxymethylcytosine (5hmC) levels (Carella et al., 2020).

Thus, besides genetic features (Brennan et al., 2013), DNA methylation/hydroxymethylation and histone modifications are major epigenetic mechanisms regulating gene expression and genomic stability whose alterations have been widely described in GBM (Hegi et al., 2005; Fernandez et al., 2018; García et al., 2018; Carella et al., 2020). In addition, the brain has a unique DNA methylation/hydroxymethylation landscape, characterized by the highest levels of 5-methylcytosine (5mC) and 5hmC of all human tissues (Nestor et al., 2012). In general terms, 5mC in promoters contributes to create a repressive chromatin environment and decreases gene expression, while 5hmC located within the body of genes may activate transcription (Chen et al., 2016). The balance between the two marks modulates a plethora of biological processes and has been extensively reported to be dysregulated in GBM (Johnson et al., 2016; López et al., 2017; Fernandez et al., 2018; García et al., 2018; Carella et al., 2020). In addition to the epigenetic marks of DNA, many studies have addressed how the modulation of chromatin structure via histone modifications is affected in the context of GBM (Liu et al., 2010). They have mostly focused on the function of histone modifying enzymes, specifically histone lysine demethylases (KDMs) and histone deacetylases (HDACs), and the use of different inhibitors (Singh et al., 2015).

However, to date little attention has been paid to the role of histone lysine methyltransferases (KMTs) in GBM (Gursoy-Yuzugullu et al., 2017). Methylation at lysine 20 of histone H4 (H4K20me) is frequently altered in cancer (Behbahani et al., 2012). However, the contribution of the epigenetic enzymes regulating these histone posttranslational modifications is still not fully understood. A preliminary genome-wide screening of candidate genes altered in GBM revealed that the gene encoding the histone methyltransferase *KMT5B* (alias *SUV420H1*) is frequently hypermethylated and hypo-hydroxymethylated in this tumor type. *KMT5B* employs H4K20me1 as a substrate, giving rise to H4K20me2 (Yang et al., 2008). In the present study we investigated the molecular basis of the deregulation of this epigenetic enzyme as well as its functional consequences and its possible tumoral role in GBM.

## Materials and Methods

### Human Brain and Glioblastoma Samples

Non-tumoral human brain (*n* = 28) and GBM samples (*n* = 37) were obtained from the Tumor Bank of the Institute of Oncology of Asturias (Asturias, Spain), the Neurological Tissue Bank of the Clinic Hospital (IDIBAPS, Barcelona, Spain), and the Biobank of the Virgen de la Salud Hospital (BioB-HVS, Toledo, Spain). Informed written consent was obtained from all patients involved in this study. Tissue collection and all analyses were conducted in accordance with the Declaration of Helsinki and approved by the Clinical Research Ethics Committee of the Principality of Asturias (Spain) (date of approval 14/10/2013, project identification code: 116/13).

### Culture of Human Glioblastoma Cell Lines and Drug Treatments

The human glioblastoma cell lines LN-18 (RRID: CVCL_0392), LN-229 (RRID: CVCL_0393), U-87MG ATCC (RRID: CVCL_0022), and T98G (RRID: CVCL_0556) were obtained and cultured according to American Type Culture Collection (ATCC) recommendations. Recently (November, 2020), all cell lines were authenticated by short tandem repeat (STR) profiling of an extracted DNA sample using AmpFLSTR^TM^ Identifiler^TM^ Plus PCR Amplification Kit (Applied Biosystems, A26182) at the Scientific and Technological Resources Unit (University of Oviedo, Asturias, Spain). Cells were grown in Dulbecco’s Modified Eagle’s Medium (DMEM) (Gibco, 41965) supplemented with 10% fetal bovine serum (FBS) (Sigma-Aldrich, F6178), 2% penicillin/streptomycin (Gibco, 15070), and 1% Amphotericin B (Gibco, 15290) at 37°C in a humidified incubator containing 5% CO_2_. Cell cultures were regularly tested and verified to be mycoplasma negative with the Mycoplasma Gel Detection kit from Biotools (Biotools, 90.022-4544), and all experiments were performed with mycoplasma-free cells. For the selection of LN-229 clones overexpressing *KMT5B*, medium was prepared that contained DMEM, 10% FBS, MEM non-essential aminoacids (Sigma-Aldrich, M7145), and Geneticin (G-418, Sigma-Aldrich, A1720) as the selection antibiotic. For the 5-aza-2′-deoxycytidine (5-AZA-dC) and vitamin C treatments, 2 × 10^6^ LN-229 cells were seeded onto P-100 plates and supplemented for 72 h with either 4 μM 5-AZA-dC (Sigma-Aldrich, A3656) alone or in combination with 50 μg/mL vitamin C (Sigma-Aldrich, A7506). Control wells contained the solvent dimethyl sulfoxide (DMSO, Sigma-Aldrich, D5879). For the *KMT5B* inhibitor A-196 treatment (Sigma-Aldrich, SML1565), 2 × 10^3^ stably-transfected LN-229 clone cells were seeded onto 8-well Lab-Tek chamber slides (Thermo Scientific, 177445) and grown for 48 h in selective medium before adding the drug (dissolved in DMSO) at 10 μM for another 48 h, with DMSO alone used in control wells.

### DNA Methylation Analysis With High-Density Array and Whole Genome Bisulfite Sequencing

Data corresponding to the oxBS and BS conversion of brain or GBM samples were obtained from our previously reported HumanMethylation 450K array (E-MTAB-6003) (Fernandez et al., 2018). Processed WGBS data were obtained from Arrayexpress (E-MTAB-5171) (Raiber et al., 2017). Both datasets were filtered to highlight the CpG coverage along the *KMT5B* gene. Estimations of 5mC and 5hmC levels were calculated with the R/bioconductor package ENmix (version 1.12.3) using the oxBS.MLE method (Xu et al., 2016).

### DNA Extraction

DNA was extracted from freshly frozen GBM, control brains and human GBM cell lines with standard phenol–chloroform-isoamyl alcohol protocol. Samples were stored at −20°C until further analysis.

### Bisulfite Pyrosequencing

Bisulfite modification of DNA was performed with the EZ DNA methylation-gold kit (Zymo Research) following the manufacturer’s instructions. The set of primers for PCR amplification and sequencing was designed using the specific software PyroMark assay design (version 2.0.01.15) (Supplementary Table 1). After PCR amplification, pyrosequencing was performed using Pyro-Mark Q24 reagents, vacuum prep workstation, equipment, and software (Qiagen).

### 5-Hydroxymethylcytosine Immunoprecipitation and Quantitative Real-Time Polymerase Chain Reaction Assay

EpiQuik Hydroxymethylated DNA Immunoprecipitation Kit (hMeDIP, Epigentek) was used for immunoprecipitation of 5hmC in 11 samples (corresponding to 5 brain samples, 5 GBM samples and LN-229 GBM cell line), according to supplier’s protocol. Input, non-specific IgG and 5hmC-enriched fractions were amplified by Quantitative Real-Time Polymerase Chain Reaction (qRT-PCR) in a StepOnePlus^TM^ Real-Time PCR machine (Applied Biosystems) with SYBR Green 2X PCR Master Mix (Applied Biosystems, 4309155) and oligonucleotides for the CpGs in the promoter of *KMT5B* listed in Supplementary Table 1. Relative 5hmC enrichment was calculated as a Fold Change relative to Input Ct Mean.

### Quantitative Real-Time Reverse Transcriptase Polymerase Chain Reaction

Total RNA was isolated from human samples and GBM cell lines using TRIzol Reagent (Life Technologies, Ref. 15596) according to the manufacturer’s instructions. To remove any possible residual genomic DNA, total RNA was treated with DNase I (Turbo DNA-free kit, Ambion-Life Technologies, Ref. AM1906). RNA was quantified both before and after DNase treatment with Nanodrop (ThermoScientific) checking purity as A260/280 ratio. cDNA was synthesized from total RNA using a SuperScript^TM^ III Reverse Transcriptase kit (Invitrogen, Ref. 18080) and following the manufacturer’s instructions. Quantitative PCR was carried out in triplicate for each sample, using SYBR Green 2X PCR Master Mix (Applied Biosystems, 4309155) and specific primers detailed in Supplementary Table 1. qRT-PCR was performed using the StepOnePlus^TM^ Real-Time PCR System (Applied Biosystems). Gene expression was normalized using *GAPDH* as endogenous control and analyzed by the comparative threshold (ΔΔCt) method.

### *KMT5B* Transfection

LN-229 cells were stably transfected with either expression vectors encoding human *KMT5B* (pEGFP-*KMT5B*) or with empty vector (pEGFP-C1), kindly provided by Professor Miki Hieda (Ehime Prefectural University of Health Sciences, Matsuyama, Japan) and constructed as described previously (Yokoyama et al., 2014), using Lipofectamine 3000 (Invitrogen, L3000015) according to the manufacturer’s instructions. Geneticin-resistant cells were selected 72 h after transfection with 1 mg/mL G-418 (Sigma-Aldrich, A-1720), after which sub-cloning was carried out by limiting dilution, and several clones overexpressing *KMT5B* were obtained. *KMT5B* expression was tested in positive clones by qRT-PCR using specific primers for *KMT5B* transcription variant 1 detection (Supplementary Table 1).

### Chromatin Immunoprecipitation Assays and Real-Time PCR Quantification (qChIP)

For real-time PCR quantification of H4K20me2-enriched genomic regions (qChIP), LN-229 cells (empty vector, *KMT5B* #3, and *KMT5B* #7) were freshly processed using the SimpleChIP Enzymatic Chromatin IP Kit with magnetic beads (Cell Signaling, 9003). Immunoprecipitations were performed using antibodies against H4K20me2 (ab9052, Abcam), total histone H3 (Abcam, ab1791) as positive control, and IgG antiserum (Abcam, ab46540) as negative control (see Supplementary Table 2). DNA was purified and used for quantitative real-time PCR with SYBR Green 2X PCR Master Mix (Applied Biosystems) and the primers for the gene loci listed in Supplementary Table 1. Input chromatin DNA was used to create a standard curve and determine the efficiency of amplification for each primer set in a StepOnePlus^TM^ Real-Time PCR machine (Applied Biosystems). All samples were measured in triplicate. IgG was used as negative control. ChIP data were analyzed and are shown in the results as the percentage relative to the input DNA amount by the equation:

$$\text{Percent}\,\text{input}=2\%\times 2^{\left[2\%\,\text{Input}\,C\,t-\text{Sample}\,C\,t\right]}$$

### Immunohistochemistry

The immunohistochemical analyses of *KMT5B* protein levels in three GBM samples and paired-normal brain tissue were performed using the EnVision FLEX Mini Kit (DAKO, K8024) and Dako Autostainer system. Briefly, paraffin embedded tissue (3–5 μm) was deparaffinized, rehydrated, and then epitopes were retrieved by heat induction (HIER) at 95°C for 20 min at pH 6.0 (DAKO, GV805) in the Pre-Treatment Module, PT-LINK (DAKO). Sections were incubated with anti-*KMT5B* antibody (sc-169462, Santa Cruz) at 1:100 dilution in EnVision^TM^ FLEX Antibody Diluent (DAKO, K8006) for 60 min after blocking endogenous peroxidase with EnVision^TM^ FLEX Peroxidase-Blocking Reagent (DAKO, DM821). Signal was detected using diaminobenzidine chromogen as substrate after incubation with Dako EnVision^TM^ FLEX/HRP (DAKO, DM822). Sections were counterstained with hematoxylin. Appropriate negative and positive controls were also tested. After the complete process, sections were dehydrated and mounted with permanent medium (Dako mounting medium, CS703). Images were captured using a Nikon Eclipse Ci microscope equipped with a 20x objective and a camera (Nikon Instruments Europe B. V.).

### Immunofluorescence

For immunofluorescence experiments 2 × 10^3^ cells were seeded onto 8-well Lab-Tek chamber slides (Thermo Scientific, 177445) and grown for 48 h. Samples were fixed with 4% formaldehyde (252931 Panreac) for 15 min at RT, and permeabilized with 1 × PBS/0.1% Triton X-100 (Sigma-Aldrich) for 20 min at RT. Blocking was performed with 1 × PBS/10% BSA (A7906 Sigma-Aldrich) at RT for 1 h, followed by incubation with the corresponding primary antibody _ rabbit anti-H4K20me1 (ab9051, Abcam) or rabbit anti-H4K20me2 (ab9052, Abcam)_ at 1:1,000 dilution (see Supplementary Table 2) in antibody diluent (EnVision FLEX DM830 Dako), for 1 h at 4°C. Secondary antibody chicken anti-rabbit IgG Alexa Fluor 488 (A-21441 Invitrogen) at 1:500 dilution was incubated for 1 h at RT protected from light. Finally, slides were mounted using EverBrite mounting medium with DAPI (23002 Biotium). Immunofluorescence images were acquired with a Zeiss microscope, equipped with a 63X/1.4 NA immersion objective and an AxioCam MRm camera (Carl Zeiss). Fluorescence intensity measurements were performed using the ZEN lite software (ZEN lite 2.3 SP1, Carl Zeiss).

### Cell Proliferation Assay

Cell proliferation rates were measured using xCELLigence Real Time Cell Analyzer (RTCA, ACEA Biosciences). Quadruplicates of mock or stably-transfected LN-229 clone cells (15 × 10^3^) were seeded onto analyzer specific plates. Cell impedance was measured every 2 h for eight consecutive days through micro-electric biosensors located at the base of the plate wells. Cell proliferation was represented by Cell Index, slope, and doubling time parameters.

### Cell Viability Assay

Cell viability of LN-229 clones stably-transfected with *KMT5B* or empty vectors was determined by 3-(4,5-Dimethylthiazol-2-yl)-2,5-Diphenyltetrazolium Bromide (MTT) assay. Briefly, ten replicates of 2 × 10^3^ cells per condition and time point (up to 72 h) were seeded onto 96-well plates. MTT (Sigma-Aldrich, M5655) was added to the corresponding wells (every 24 h for 3 days) to a final concentration of 500 μg/mL, and incubated at 37°C, 5% CO_2_ for 3 h. The MTT was then removed and the formazan crystals formed were dissolved in DMSO (Sigma-Aldrich-Aldrich, D5879). Absorbance at 570 nm was measured with an automated microtiter plate reader (Synergy HT, BioTek).

### Colony Formation Assay

Colony formation assays of LN-229 clones stably-transfected with *KMT5B* or empty vectors were conducted as described by Franken et al. (2006), with minor modifications. Briefly, cells were seeded at two different densities (100 and 200 cells) onto P-6 wells. After incubation for 14 days, stable colonies were fixed and stained with a mixture of 6.0% glutaraldehyde (Sigma-Aldrich, 340855) and 0.5% crystal violet (Sigma-Aldrich, C3886). After washing, the number of colonies was counted manually. The results were expressed as mean Plating Efficiency (PE), a parameter which represents the ratio between the number of grown colonies versus the number of cells seeded at each different density.

PE=[Numberofcoloniesformed÷Numberofcellsseeded]×100

### Cell Cycle Analysis

Cell cycle analysis of LN-229 clones stably-transfected with *KMT5B* or empty vectors was conducted by flow cytometry using propidium iodide (PI, Sigma-Aldrich, P4170). Briefly, cells were trypsinized, washed with PBS, and fixed with cold absolute ethanol overnight at −20°C. Cells were then washed twice with PBS and dyed with PI, and RNase A for 30 min at 37°C in the dark. The suspension was filtered with a nylon mesh filter and analyzed using a BD FACS Aria Ilu cytometer (BD Biosciences) and FlowJoV10 software.

### Tumor Xenografts

For the xenograft experiments, 5-weeks old NU/NU female mice (*n* = 4) were purchased from Charles River Japan Inc. (Kanagawa, Japan). Animal housing and experimental procedures were approved by the Animal Ethics Committee of University of Oviedo (Asturias, Spain) on 21/06/2018 with the project identification code PROAE 18/2018. Mice were subcutaneously injected in each flank with 1 × 10^6^ LN-229 cells (mock and *KMT5B* stably-transfected clones) suspended in culture medium 1:1 with Matrigel (Corning, Ref 354248). Tumor volumes were measured with a calliper twice a week and calculated using the formula *V* = 4/3π (*Rr*)^2^, were *R* is the maximum diameter and *r* is the minimum diameter. After animal sacrifice, the tumors were excised and weighed.

### RNAseq

For RNA-seq, total RNA from three replicates of mock and *KMT5B* stably-transfected clones was isolated using the RNeasy Mini Kit (Cat. No. 74104, Qiagen) according to the manufacturer’s instructions, submitted to GeneWiz (Germany) for next-generation sequencing purposes, and to the European Nucleotide Archive (ENA) database (accession number PRJEB39613). Library preparation was performed using the Illumina Poly(A) selection method according to GeneWiz pipeline for standard RNA sequencing. Samples were sequenced using the Illumina HiSeq platform using 2 × 150 bp configuration mode. Adaptor removal was performed using fastp (v. 0.20.1), and the filtered reads were aligned to the human reference genome GRCh38 using the ultra-fast selective-alignment provided by SALMON (v.1.2.1). A prior step was introduced to generate the gentrome files from the concatenated GENCODE transcriptome and GRCh38 genome primary assembly (V29). The sequencing coverage and quality statistics for each sample are summarized in Supplementary Table 6. Differential gene expression analyses were conducted using the DESeq2 R/Bioconductor package (v.1.22.2) using as input the read count matrices obtained from SALMON. Genes with an absolute Log2 fold change = 1 and FDR < 0.05 were considered to be significant for downstream purposes. Subsequent enrichment analyses were performed using the R/Bioconductor package clusterProfiler (v.3.16.0). Significant gene-disease associations were represented using the R/Bioconductor package enrichplot (v.1.8.1).

### Statistical Analysis

All statistical analyses were conducted using the R statistical programming language (version 3.4.0). Specific analyses are described in the corresponding section.

## Results

### *KMT5B* Shows Altered 5mC/5hmC Pattern in GBM

**FIGURE 1:**
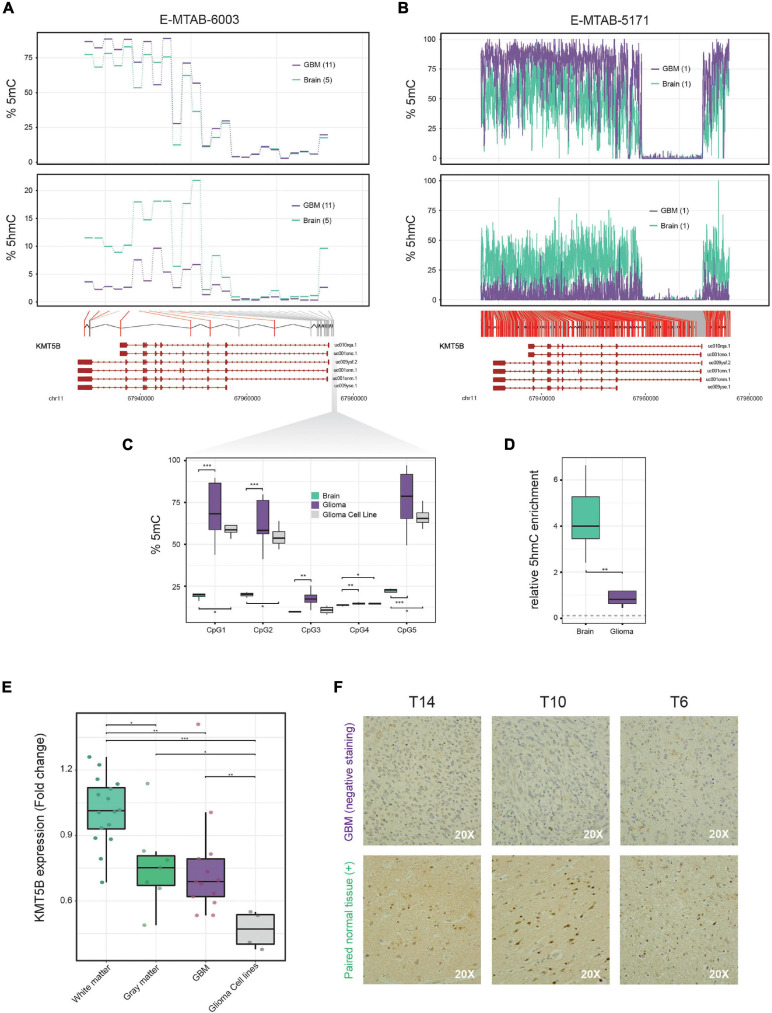
5mC and 5hmC profiling along the *KMT5B* locus in brain and glioblastoma multiforme (GBM) samples and analysis of *KMT5B* expression in brain, GBM and GBM cell lines. **(A)** Line plots represent percentage of average 5mC (*upper panel*) and 5hmC values (*lower panel*) corresponding to 25 CpG positions in the *KMT5B* gene for five control brain and eleven GBM samples, depicted in blue and violet, respectively. Data were obtained from a genome-wide Illumina HumanMethylation 450 K array (E-MTAB-6003). Welch *t*-tests were applied for each CpG and those that were significant are depicted in red (*q*-value < 0.05). **(B)** WGBS dataset with high content profiling of 5mC and 5hmC (accession number E-MTAB-5171) confirmed the altered 5mC/5hmC pattern of *KMT5B*. CpGs depicted in red represent those with changes = 15%. Lower diagrams in panels **(A,B)** represent the relative location of the analyzed CpG sites along *KMT5B* locus. **(C,D)** Biological validation of the methylation and hydroxymethylation values in five *KMT5B* promoter CpGs using, respectively, bisulfite pyrosequencing and hMeDIP. The gray dotted line in panel **(D)** indicates 5hmC levels in GBM cell line LN-229. Wilcoxon rank sum tests were applied (*p < 0.05; **p < 0.01; ***p < 0.001). **(E)** Box plot represents *KMT5B* mRNA relative expression in 16 white matter and 7 gray matter control brains, 13 GBM samples and 4 GBM cell lines, measured by qRT-PCR in relation to *GAPDH* and represented as Fold Change relative to white-matter control brains. Wilcoxon rank sum tests were applied (*p < 0.05; **p < 0.01; ***p < 0.001). **(F)** Representative images of *KMT5B* protein levels detected in GBM samples and their paired non-tumoral brain tissue by immunohistochemistry (IHC), shown at 20× magnification. Only non-tumoral brain sections showed positive *KMT5B* nuclear staining.

On the basis of data from our previously reported 450 K Illumina methylation array of 11 GBM and 5 non-tumoral brain samples (Fernandez et al., 2018), we performed a genome-wide screening of candidate genes which are altered in GBM. We found that *KMT5B*, a gene encoding a histone methyltransferase, had altered DNA methylation and hydroxymethylation patterns. Specifically, compared to healthy brain tissue, GBM showed higher levels of 5mC and greatly reduced levels of 5hmC in the CpGi at the promoter and several CpG sites within the *KMT5B* gene body (Figure 1A and Supplementary Table 3). Comparison with publicly available whole genome bisulfite sequencing (WGBS) data, with a deeper CpG coverage along the *KMT5B* locus (Raiber et al., 2017), confirmed a 5mC/5hmC imbalance in GBM as compared to non-tumoral brain (Figure 1B and Supplementary Table 4).

We validated 5mC array data by means of the bisulfite pyrosequencing of five representative CpG sites at *KMT5B* promoter in the same specimens included in the array, as well as in a larger set of samples consisting of: 27 GBM, 4 non-tumoral brains, and 4 human GBM cell lines. Pyrosequencing results confirmed the 5mC pattern observed in the array (Figure 1C and Supplementary Table 5). With respect to 5hmC array data, we validated those results using a different technique that relied not on oxidative bisulfite conversion, but on DNA immunoprecipitation with an antibody against 5hmC. We used an hMeDIP Kit (see “Materials and Methods” section) with 5 GBM, 5 non-tumoral brain samples and the human GBM cell line LN-229. qRT-PCR was used to analyse the samples and the results confirmed the extensive loss of 5hmC affecting the *KMT5B* locus in GBM (Wilcoxon rank sum test, ^∗∗^*p* < 0.01; Figure 1D).

### *KMT5B* Expression Is Downregulated in a Subset of Human GBM Samples and in GBM Cell Lines

Next, we wanted to investigate whether the aberrant methylation landscape of *KMT5B* could have an effect at the transcriptional regulation level. In order to determine the expression status of *KMT5B* in GBM, we measured its mRNA levels by means of qRT-PCR in 13 GBM samples, four human GBM cell lines, and 23 non-tumoral control brains (seven corresponding to gray matter and 16 to white matter samples). The results showed that *KMT5B* mRNA expression was significantly downregulated in a subset of GBM samples compared with non-tumoral brains (Figure 1E). Comparison with gene expression array data obtained from different brain datasets in ArrayExpress (Günther et al., 2008; Sim et al., 2009; Grzmil et al., 2011) confirmed that downregulation of *KMT5B* affects a subset of GBMs (Supplementary Figure 1, Supplementary Table 8, and Supplementary Methods).

To validate our observations at the protein level, we subjected three GBM samples and their paired non-tumoral brain tissue to immunohistochemistry (IHC). The results were in line with the mRNA expression data and revealed that *KMT5B* protein expression was high in non-tumoral brain tissues and low to absent in GBM (Figure 1F).

These findings point to a potential epigenetic silencing of *KMT5B* through the methylation of the promoter and hypo-hydroxymethylation of the gene body in GBM.

### Correlation Between Epigenetic Regulation and *KMT5B* Gene Expression

**FIGURE 2:**
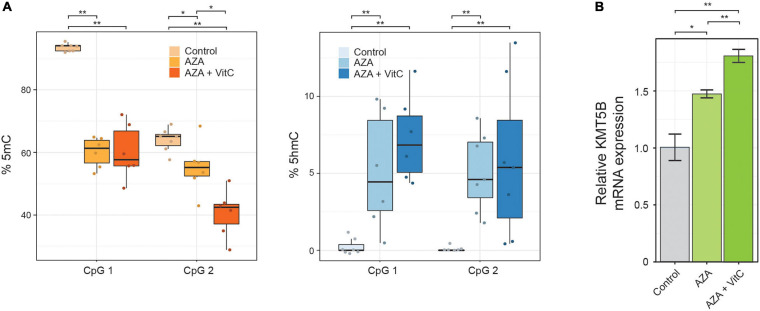
Pharmacological restoration of 5mC/5hmC levels in human GBM cell line LN-229. Cells were treated with demethylating agent 5-AZA-dC, either alone or in combination with vitamin C (Vit C). The effects on 5mC/5hmC levels and on *KMT5B* expression were assessed by **(A)** bisulfite pyrosequencing at two intragenic CpG positions (Wilcoxon rank sum test; *p < 0.05; **p < 0.01) and **(B)** *KMT5B* mRNA relative expression by means of qRT-PCR. The data are the average ±SEM of three independent experiments (Welch *t*-test; *p < 0.05; **p < 0.01).

To further explore the possible functional link between the epigenetic alterations described above and *KMT5B* repression, we performed a pharmacological restoration of 5mC/5hmC levels in human GBM cell line LN-229. To this end, we incubated LN-229 cells with 5-aza-2′-deoxycytidine (5-AZA-dC) either alone or together with vitamin C. 5-AZA-dC is an epigenetic drug, widely used in clinical practice, that decreases 5mC levels through DNA methyl-transferases (DNMTs) inhibition, and increases 5hmC levels through upregulating the expression of Ten-Eleven-Translocation proteins (TETs) (Sajadian et al., 2015). Vitamin C, a TET cofactor, has been shown to increase intragenic 5hmC levels and to act synergistically with 5-AZA-dC *in vitro* (Sajadian et al., 2016). The treatments carried out here caused a significant decrease in 5mC and an increase in 5hmC levels, as confirmed at two intragenic CpG sites (CpG in the array CG27086672 and the next downstream CpG position) by means of bisulfite pyrosequencing (Wilcoxon rank sum tests, ^∗^*p* < 0.05; ^∗∗^*p* < 0.01; Figure 2A and Supplementary Table 5). At the mRNA level, *KMT5B* expression was also upregulated, especially when both drugs were present (Welch *t*-test, ^∗∗^*p* < 0.01; Figure 2B).

Taken together, these data indicate that aberrant epigenetic regulation contributes to *KMT5B* repression in GBM.

### Ectopic Expression of *KMT5B* Reduces the Tumorigenic Potential of Glioblastoma Cells *in vitro*

**FIGURE 3:**
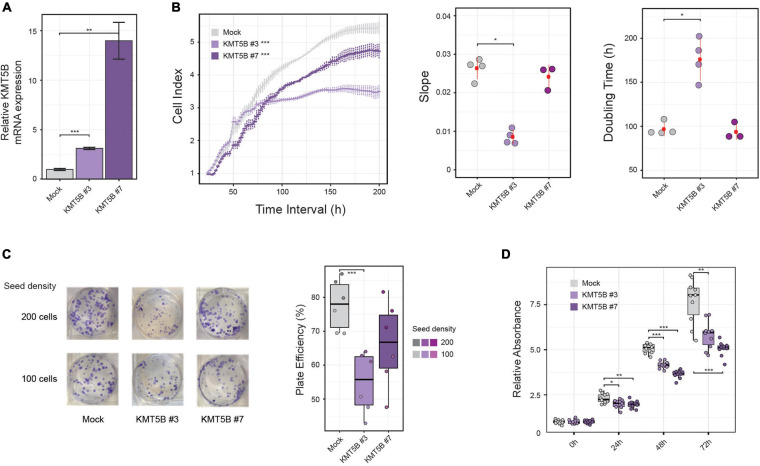
*In vitro* effect of *KMT5B* ectopic expression. Human GBM cell line LN-229 was transfected with an empty (control) or a *KMT5B* expression vector. **(A)** Stable transfectants were obtained after selection with 1 mg/mL G-418, and named mock, *KMT5B* #3, and *KMT5B* #7. *KMT5B* expression was confirmed by qRT-PCR (the data shown are the average ±SEM of three independent experiments. Welch *t*-test; **p < 0.01; ***p < 0.001). **(B)** The proliferation rate of mock and the two *KMT5B* stably-transfected clones was measured by an impedance-based assay and represented as cell index units [General linear model (GLM) ***p-value < 0.001], slope and doubling time for three to four technical replicates (Wilcoxon rank sum tests; *p-value < 0.05). **(C)** On the left, representative image of plates showing colony formation assay. Experiments were done in triplicate for each clone and seeding density. Results are represented in the box plot on the right as plate efficiency (Wilcoxon rank sum tests; ***p-value < 0.001). **(D)** Cell viability was determined by MTT assay on control and *KMT5B* transfected cells. Box plot represents the average absorbance of ten replicates at several time points, relative to the absorbance at time 0 h for each clone (Wilcoxon rank sum test; *p < 0.05; **p < 0.01; ***p < 0.001).

To delve into the possible role of *KMT5B* epigenetic repression in GBM, we stably transfected either a *KMT5B* expression plasmid or an empty vector (mock) into the human GBM cell line LN-229, which exhibits hypo-hydroxymethylation and downregulation of *KMT5B*. Two clones, namely *KMT5B* #3 and *KMT5B* #7, were selected among those that were drug resistant, and the expression of *KMT5B* was confirmed by qRT-PCR (Figure 3A). Impedance-based proliferation assays showed that *KMT5B*-transfected clones grew less than control cells (Figure 3B). Overexpression of *KMT5B* also reduced the clonogenicity of the clones compared with the mock transfected cells (Figure 3C). In addition, an MTT assay demonstrated that *KMT5B* overexpression caused a significant decrease in cell viability (Figure 3D). Cell cycle analysis confirmed that *KMT5B* overexpression induced G2/M arrest, as was demonstrated previously by Evertts et al. (2013), thus explaining the decreased cell proliferation and viability of the overexpressing clones (Supplementary Figure 2).

**FIGURE 4:**
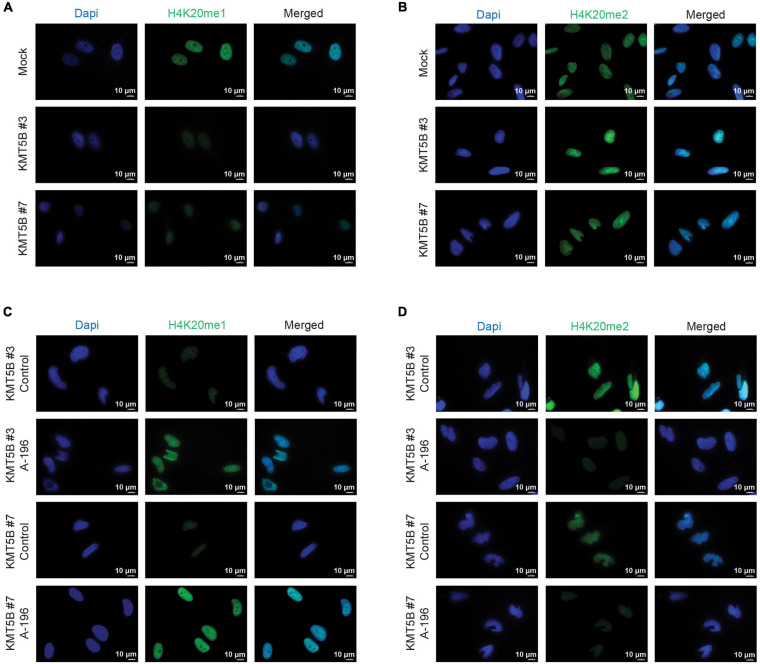
Immunofluorescence analysis of mono- and dimethylation of H4K20. Representative images of mock and *KMT5B*-transfected clones immunostained with **(A)** anti-H4K20me1 or **(B)** anti-H4K20me2 antibodies (green). In panels **(C,D)** representative images of *KMT5B*-transfected clones immunostained with **(C)** anti-H4K20me1 or **(D)** anti-H4K20me2 antibodies (green) after treating the cells with *KMT5B* inhibitor A-196 for 48 h. Nuclei were counterstained with DAPI (blue). Bars: 10 μm.

As expected, the increased expression of *KMT5B* elevated the levels of its histone mark H4K20me2, concomitantly reducing those of H4K20me1, as seen by immunofluorescence (IF) in stably-transfected clones versus mock (Figures 4A,B and Supplementary Figures 3A,B). Conversely, inhibition of *KMT5B* enzymatic activity with the selective drug A-196 (Bromberg et al., 2017) increased monomethylated and decreased dimethylated forms of H4K20 in stably-transfected clones, thus recapitulating the histone H4K20 methylation landscape of LN-229 GBM cell line (Figures 4C,D and Supplementary Figures 3C,D).

### *KMT5B* Overexpression Reduces Tumor Growth in Nude Mice

**FIGURE 5:**
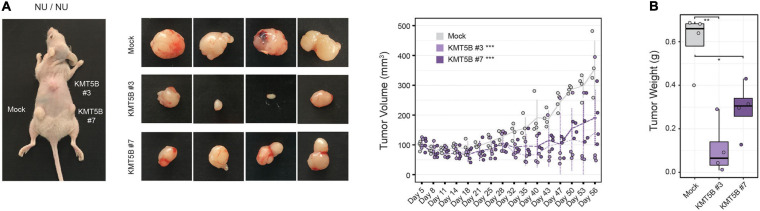
*In vivo* effect of *KMT5B* overexpression. **(A)** Mock or *KMT5B*-transfected LN-229 cells were subcutaneously injected into the flanks or the neck base of 4 nude mice. An image of the tumors excised after animal sacrifice is shown on the left. Tumor growth was monitored for 56 days [General linear model (GLM) ***p-value < 0.001]. **(B)** Box plot represents average tumor weights for the tumors generated by each clone (Wilcoxon rank sum tests; *p < 0.05; **p < 0.01).

In light of the above data, we sought to further characterize the role of *KMT5B* expression in GBM *in vivo*. To this end, we subcutaneously injected stably-transfected *KMT5B* or mock LN-229 cells into nude mice. Tumor volume was notably smaller in mice flanks receiving *KMT5B*-overexpressing LN-229 clones compared to those injected with empty vector transfected cells (General linear model, ^∗∗∗^*p*-value < 0.001; Figure 5A). At the time of sacrifice, 56 days after tumor-xenograft implantation, the average weight of tumors generated by *KMT5B*-overexpressing clones was significantly lower than those from control cells (Wilcoxon rank sum tests; ^∗^*p* < 0.05; ^∗∗^*p* < 0.01; Figure 5B). The tumor xenografts thereby revealed that *KMT5B* overexpression also slows tumorigenesis *in vivo*, suggesting that *KMT5B* could play a possible role as a putative tumor suppressor in GBM.

### Overexpression of *KMT5B* Affects Genome-Wide Gene Expression in GBM Cells

**FIGURE 6:**
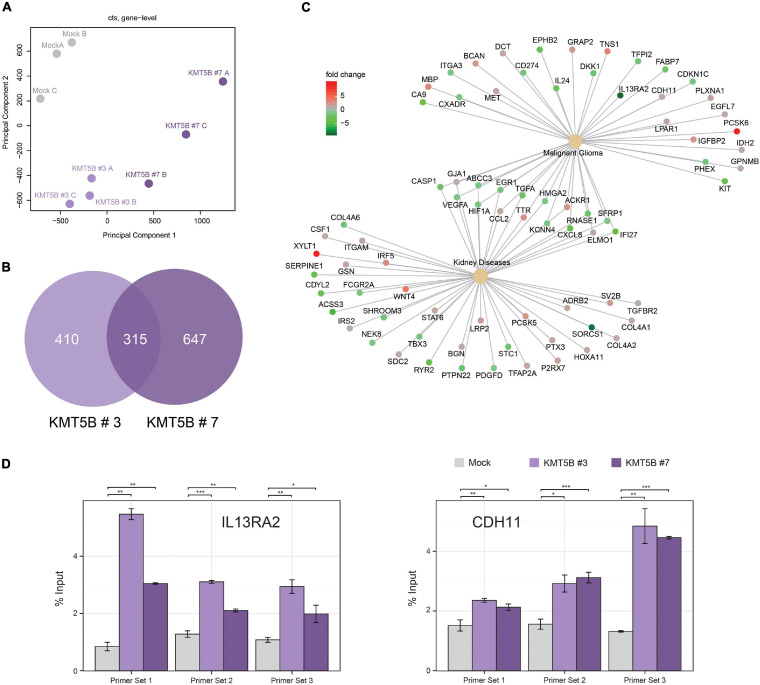
Overexpression of *KMT5B* alters the expression of several genes, including *IL13RA2.* **(A)** Principal component analysis of the three replicates of each clone subjected to RNAseq **(B)** Venn diagram showing differentially expressed genes in *KMT5B*-expressing clones *KMT5B*#3 and *KMT5B*#7. Both comparisons were made independently against the expression profile of the LN-229 control cells (mock). **(C)** Network diagram showing the functional relationships between the genes differentially expressed in the RNAseq analysis and disease ontologies (glioblastoma and renal diseases) obtained from DisGeNET. To facilitate the interpretation of the data, the relationships of genes and ontologies common to both conditions are indicated. Information on the expression levels of the different genes compared to the control (mock) is indicated by the colour scale. **(D)** Enrichment of H4K20me2 upstream of the *IL13RA2* and *CDH11* transcription start sites due to *KMT5B* overxpression. Chromatin immunoprecipitation (ChIP) assays were performed in mock and *KMT5B* stably-transfected LN-229 cells using either anti-H4K20me2 polyclonal antibody or control IgG (see Supplementary Table 2). DNA eluted from the ChIP assay was amplified by qRT-PCR with primer sets mapping to *IL13RA2* or *CDH11* promoters (listed in Supplementary Table 1). The enrichment of H4K20me2 at the indicated regions in *KMT5B* clones was compared to the same regions in mock control cells (Welch *t*-tests; *p < 0.05; **p < 0.01; ***p < 0.001).

In an attempt to shed light on the molecular pathways and mechanisms by which *KMT5B* decreases GBM tumorigenesis, we subjected our stably-transfected overexpressing or mock LN-229 clones to RNAseq (Supplementary Table 6). *KMT5B* has been proposed to impact transcriptional regulation by means of its product H4K20me2 (Miao and Natarajan, 2005; Vakoc et al., 2006). Our results showed that overexpression of *KMT5B* changed the transcriptome of GBM cells through up- or downregulation of hundreds of genes (Figure 6 and Supplementary Table 7). Specifically, for clone *KMT5B*#3 a total of 725 genes were significantly affected, corresponding to 333 upregulated and 392 downregulated ones. Regarding *KMT5B*#7, expression changes implicated a total of 962 genes of which 484 were upregulated and 478 downregulated. The Venn diagram in Figure 6B represents the overlap of differentially expressed genes in *KMT5B* clones as compared to the LN-229 control cells (mock). The two clones shared a total of 315 differentially expressed genes. Among the upregulated ones, *CDH11* (*cadherin-11*) has been associated with blocking the invasion of GBM cells *in vitro* (Delic et al., 2012). Interestingly, several of those genes had been previously described to be overexpressed and implicated in GBM tumorigenesis, including: *CA9* (*carbonic anhydrase 9*) (Boyd et al., 2017), *PDL1* (*Programmed Death Ligand 1*, also known as *CD274*) (Jacobs et al., 2009), and *IL13RA2* (*Interleukin 13 Receptor Subunit Alpha 2*). We focused on *IL13RA2* as this gene encodes a monomeric IL4-independent and high grade glioma-associated IL13 receptor (Mintz et al., 2002). IL13RA2 is overexpressed in most patients with GBM but not in normal brain (Brown et al., 2013). As expected, the gene-disease associations platform DisGeNET related the above-mentioned genes with malignant glioma. Interestingly, it also highlighted the association of certain other genes with both malignant glioma and kidney diseases, thus pointing to a yet-undescribed hypothetical role of *KMT5B* in such renal pathologies (Figure 6C).

To verify the RNAseq results and assess the involvement of *KMT5B* and H4K20 methylation in the regulation of *IL13RA2* and *CDH11* expression, we performed a ChIP assay for H4K20me2 in our clones. The analysis of immunoprecipitated DNA by qRT-PCR demonstrated that H4K20me2 is enriched immediately upstream of the *IL13RA2* and *CDH11* transcription start sites in the *KMT5B*-overexpressing clones, but not in mock-transfected GBM cells (Figure 6D). Additionally, we conducted pairwise correlation analyses between *KMT5B* and candidate genes using the cBioPortal platform (Cerami et al., 2012) with microarray data obtained from the comprehensive characterization of glioblastoma performed by the TCGA consortium (Cancer Genome Atlas Research Network, 2008). Considering all the samples from the GBM dataset (*n* = 206), *KMT5B* mRNA expression showed inverse correlation with *IL13RA2* and direct correlation with *CDH11*, which is in line with our RNAseq and qChIP results (Supplementary Figure 4, upper panels). When grouping the samples by their corresponding GBM gene expression profile (Classical, Mesenchymal, Neural, and Proneural), we observed a similar trend in correlations across all subtypes, and they were particularly significant in the neural GBM subtype (Supplementary Figure 4). Altogether, these data suggest that *IL13RA2* and *CDH11* could represent direct targets of *KMT5B*-mediated gene expression regulation.

## Discussion

The involvement of KMTs in GBM pathogenesis has not as yet been fully elucidated. Referring back to our data from a 450K Infinium Illumina methylation array reported in 2018 (Fernandez et al., 2018), we found that the *KMT5B* gene shows an altered DNA methylation and hydroxymethylation profile in GBM as compared to non-tumoral brain samples. The present study aimed to investigate the contribution of this chromatin modifying gene to the etiology of GBM. We obtained evidence that it could exert an anti-tumoral effect, likely through its histone mark H4K20me2.

Expression analyses revealed that *KMT5B* hypermethylation and hypo-hydroxymethylation correlated with its decreased expression in some of our GBM specimens in comparison with normal brain tissue strongly indicating that the epigenetic downregulation of *KMT5B* might be relevant for a subset of GBM tumors. In fact, gene inactivation by DNA hypermethylation of CpGis at gene promoters is a well-established epigenetic hallmark in cancer (Koch et al., 2018). As for DNA hydroxymethylation, its presence at gene bodies is positively correlated with gene expression (Jin et al., 2011b), while its loss has been described in several cancers, including GBM (Jin et al., 2011a). This was reinforced in this work through the pharmacological reestablishment of 5mC and 5hmC levels, using a DNMTs inhibitor and a TETs cofactor, which successfully rescued *KMT5B* expression in our human GBM cell line LN-229. Nevertheless, unlike Sajadian et al. (2016), we did not find differences in the magnitude of changes in 5mC and 5hmC levels using 5-AZA-dC alone or when in combination with vitamin C. This apparent discrepancy might be explained by the fact that we specifically evaluated the methylation/hydroxymethylation state of two intragenic CpGs rather than considering global levels of those marks as in the study cited.

At the functional level, overexpression *KMT5B* reduced cell proliferation, cell viability and clonogenic potential *in vitro*, and tumor growth *in vivo* in mice xenografts. Interestingly, the loss of proliferative capacity induced by *KMT5B* overexpression in our clones was not related to apoptotic mechanisms, but rather to a G2/M cell cycle blockade. This is in agreement with a previous study showing that *KMT5B* overexpression causes G2 arrest (Evertts et al., 2013), and is consistent with the widely described role of methylated forms of H4K20 in cell cycle progression (Pesavento et al., 2008).

Regarding the histone mark deposited by *KMT5B*, H4K20me2, its levels were confirmed to be diminished in mock-transfected GBM cells, and to recover upon *KMT5B* expression in stably-transfected clones, as demonstrated through immunohistochemical analyses. Concurrently, levels of the histone mark H4K20me1, which serves as *KMT5B* substrate, varied according to the expression of this enzyme, i.e., they were very low in *KMT5B*-expressing clones and higher in mock-transfected ones. We further confirmed the H4K20 patterns by treating the cells with A-196, a drug which causes *KMT5B* enzymatic inhibition. Indeed, the treatment reverted the levels of both marks in *KMT5B* stably-transfected clones, increasing H4K20me1 and decreasing H4K20me2 to a comparable extent as seen in LN-229 GBM cells.

Furthermore, ectopic expression of *KMT5B* was associated with transcriptional changes of several GBM-related genes, as seen by the results of RNAseq analyses. The role of H4K20 monomethylation in gene expression has been controversial for some time due to studies associating this mark with both transcriptional repression (Nishioka et al., 2002) and activation (Talasz et al., 2005; Vakoc et al., 2006). However, more recent works and all the genome-wide ChIP analyses to date have rendered compelling results linking this mark with transcription activation (Barski et al., 2007; Li et al., 2011) and, indeed, it is one of the histone modifications that is most strongly correlated with active transcription (Barski et al., 2007; Karliæ et al., 2010; Veloso et al., 2014). By contrast, to date, the function of H4K20 dimethylation in gene expression has scarcely been addressed. That said, studies carried out in human cells suggest that H4K20me2 could be related to gene silencing (Miao and Natarajan, 2005; Vakoc et al., 2006; Gwak et al., 2016). The results presented here highlight the importance of *KMT5B* epigenetic downregulation in GBM, which leads to a distorted H4K20 methylation pattern. This is in line with the roles for H4K20me1 and H4K20me2 in gene expression mentioned above, and tie in with the concept of epigenetic de-repression of oncogenes. While it is well-known that the epigenetic silencing of tumor suppressor genes occurs during tumorogenesis, the role of epigenetic de-repression of oncogenes due to the loss of repressive chromatin marks has remained partially unexplored (Baylin and Jones, 2016). Our experiments showed that the epigenetic downregulation of *KMT5B* impairs the balance between H4K20me species in human GBM cell line LN-229. In turn, this may cause global transcriptomic changes, given our finding that overexpression of *KMT5B* restored H4K20me2 patterns and decreased the expression of several oncogenes, such as *IL13RA2*, a tumor-restricted receptor (Mintz et al., 2002). IL13 is a pleiotropic Th2-derived cytokine implicated in inflammation and immunomodulation, and is known to induce apoptosis in different cell types, including GBM (Hsi et al., 2011). IL13RA2 binds IL13 with a higher affinity than the physiological IL13RA1/IL4RA receptor but prevents apoptosis as it does not transduce the signal for STAT6 pathway activation (Kawakami et al., 2001). Although the functional significance of IL13RA2 expression is not fully understood, it is considered an inhibitory or decoy receptor in GBM (Rahaman et al., 2002) that contributes to tumor growth (Hsi et al., 2011) and several studies have found that its overexpression is associated with higher glioma grades and poor patient prognosis (Brown et al., 2013; Han and Puri, 2018). Investigations in pancreatic, colorectal, and ovarian cancer have shown that IL13RA2 overexpression promotes tumor migration and invasion (Fujisawa et al., 2009, 2012; Barderas et al., 2012). More recent studies in GBM have also pointed to a role for IL13RA2 in stimulating cell growth and metastasis (Tu et al., 2016). As such, IL13RA2 has become an attractive therapeutic target in GBM, even though the regulatory mechanisms of its expression are currently unknown (Sharma and Debinski, 2018). By means of RNAseq and ChIP experiments, our study suggests that *KMT5B* and its mark H4K20me2 might be involved in IL13RA2 regulation since overexpression of *KMT5B* in our clones caused H4K20me2 deposition upstream of the *IL13RA2* promoter and a concomitant downregulation of *IL13RA2* mRNA.

Notwithstanding, we observed that some genes were upregulated upon *KMT5B* overexpression. Among them *CDH11*, which encodes Cadherin 11, has been previously reported to block GBM cell invasion *in vitro* (Delic et al., 2012). One plausible explanation is that H4K20me2 might function as both transcriptional activator or repressor depending on the cellular context, in concert with other associated histone modifications and readers. That dual role on gene expression modulation would fit with the abundance and ubiquitous distribution of this mark on chromatin (Pesavento et al., 2008; Schotta et al., 2008). Future research is guaranteed in order to elucidate the context dependant function of H4K20me2. Consequently, it is possible that the anti-tumoral effect of *KMT5B* observed in our study would not be due to the overexpression of this epigenetic modifier itself, but rather might be mediated by the re-establishment of levels of its product H4K20me2 in the promoter region of key GBM-related genes, such as *IL13RA2* and *CDH11*.

The dynamic and reversible nature of H4K20me2 opens the possibility of targeting its demethylase (KDM), via small-molecule inhibitors, to restore this histone mark in GBM. Indeed, in recent years, histone modifiers, such as KDMs, are envisaged as attractive druggable targets for GBM therapy (Jones et al., 2016). Although H4K20me2 is the most abundant form of H4K20 in eukaryotic cells (Pesavento et al., 2008), only a H4K20me2 demethylase has been described so far. It is LSD1n, a neuron-specific splicing variant of the flavin-dependent monoamine oxidase LSD1 with acquired new specificity against H4K20me2 and H4K20me1 (Wang et al., 2015). However, caution is needed when considering LSD1n as a potential therapeutic target using a hypothetical specific KDM inhibitor. On the one hand, there is increasing evidence that histone modifications do not act alone, but influence one another. Hence, modulation of H4K20me2 levels via a LSD1n inhibitor could potentially affect the modification status of other histone residues on the vicinity with unknown effects. On the other hand, non-histone targets of LSD1n should also be taken into account. Moreover, it has been reported that LSD1n promotes neuronal activity-regulated gene expression and plays a critical role in neurite morphogenesis, spatial learning, long-term memory formation, and modulation of emotional behavior (Zibetti et al., 2010; Wang et al., 2015; Rusconi et al., 2016). All of the above reveals the challenging pleiotropic consequences and the complexity of the epigenetic regulation via KDM inhibitors within CNS. In the future, a deeper understanding of the roles and LSD1n targets other than histones, as well as the transcriptional networks regulated by H4K20me2, or even the discovery of other yet unknown specific demethylases, could boost the development of novel specific KDM inhibitors and inform novel strategies in cancer therapy.

Taken together, our results suggest that when *KMT5B* expression and its histone mark H4K20me2 are perturbed in GBM through deregulated epigenetic mechanisms, this process has a genome-wide effect on gene transcription and may contribute to malignant transformation.

## Data Availability Statement

The datasets presented in this study can be found in online repositories. The names of the repository/repositories and accession number(s) can be found below: ENA, PRJEB39613.

## Ethics Statement

The studies involving human participants were reviewed and approved by Clinical Research Ethics Committee of the Principality of Asturias (Spain). The patients/participants provided their written informed consent to participate in this study. The animal study was reviewed and approved by Animal Ethics Committee of University of Oviedo (Asturias, Spain).

## Author Contributions

MF and AF: conceptualization, study supervision, and funding acquisition. VL, JT, AC, MG, PS-O, RP, CM, RU, AiA, and DN: development of methodology. VL, MC-T, AuA, MM, and BM: acquisition of data. VL, JT, AF, and MF: analysis and interpretation of data, and writing–review and editing. JT and RP: software analysis. VL: writing–original draft preparation. MF: final approval. All authors have read and agreed to the published version of the manuscript.

## Conflict of Interest

The authors declare that the research was conducted in the absence of any commercial or financial relationships that could be construed as a potential conflict of interest.

## Publisher’s Note

All claims expressed in this article are solely those of the authors and do not necessarily represent those of their affiliated organizations, or those of the publisher, the editors and the reviewers. Any product that may be evaluated in this article, or claim that may be made by its manufacturer, is not guaranteed or endorsed by the publisher.

## Abbreviations

5-AZA-dC: 5-aza-2 ′-deoxycytidine
5hmC: 5-hydroxymethylcytosine
5mC: 5-methylcytosine
ATCC: American Type Culture Collection
CFA: colony formation assay
ChIP: chromatin immunoprecipitation
CpGi: CpG island
DMEM: Dulbecco’s modified Eagle’s medium
DMSO: dimethyl sulfoxide
DNMTs: DNA methyltransferases
ENA: European Nucleotide Archive
FBS: fetal bovine serum
GBM: glioblastoma multiforme
GLM: general linear model
H4K20me: methylation at lysine 20 of histone H4
HDACs: histone deacetylases
hMeDIP: 5-hydroxymethylcytosine immunoprecipitation
KDMs: histone lysine demethylases
KMTs: histone lysine methyltransferases
MTT: 3-(4,5-dimethylthiazol-2-yl)-2,5-diphenyltetrazolium bromide
PCR: polymerase chain reaction
PE: platting efficiency
PI: propidium iodide
RTCA: real time cell analysis system
qRT-PCR: quantitative real-time reverse transcriptase polymerase chain reaction
SAHA: suberoylanilide hydroxamic acid
STR: short tandem repeat
TET: ten–eleven translocation
TMZ: temozolomide
Vit C: vitamin C
WGBS: whole genome bisulfite sequencing.
