# Supplementary figures and images for: Epigenetic Deregulation of the Histone Methyltransferase KMT5B Contributes to Malignant Transformation in Glioblastoma
Source: Front Cell Dev Biol. 2021 Aug 10;9:671838. doi: 10.3389/fcell.2021.671838 (PMC8383299; doi:10.3389/fcell.2021.671838)

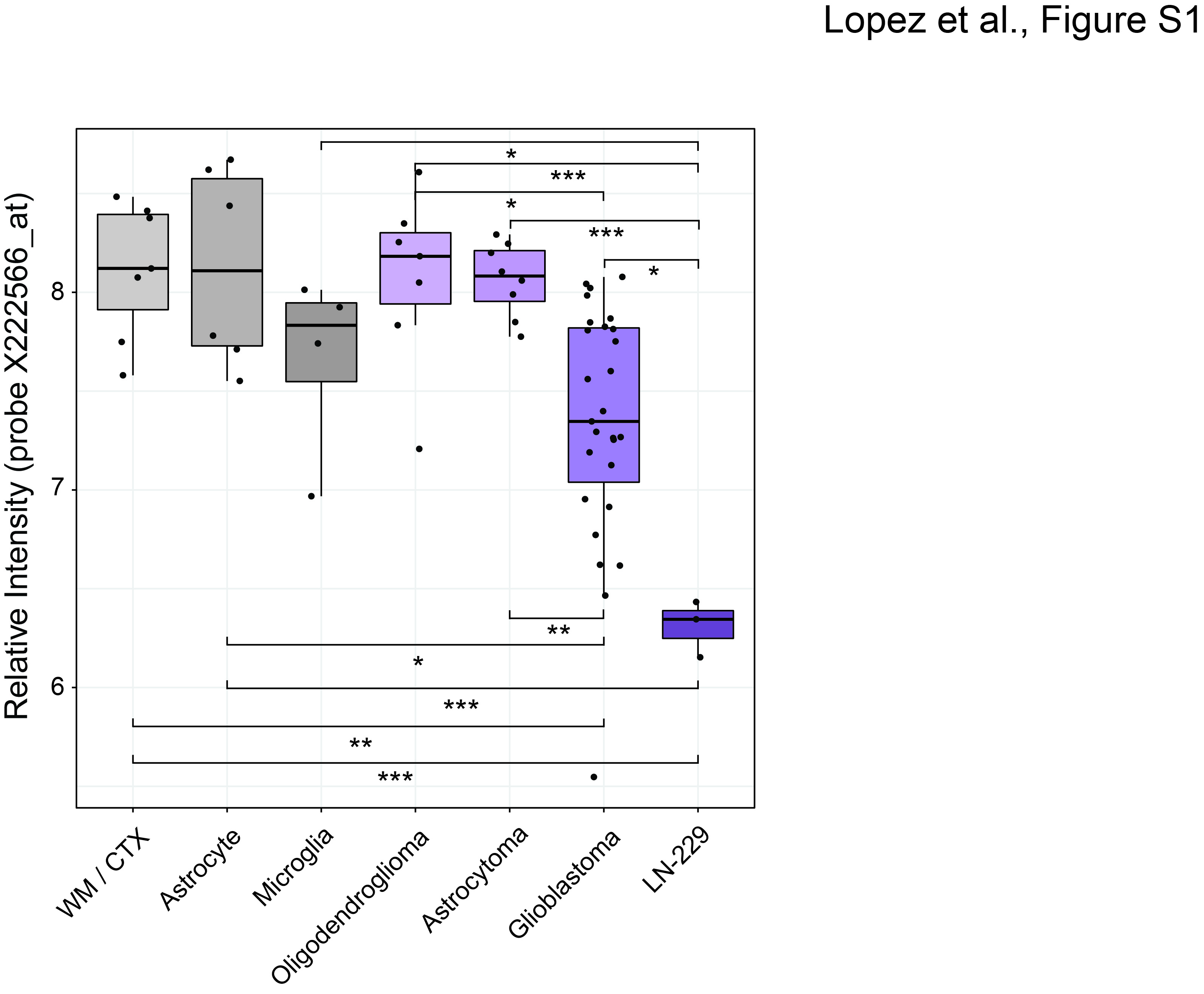

Supplement: Supplementary file 1 [file Image_1.JPEG]

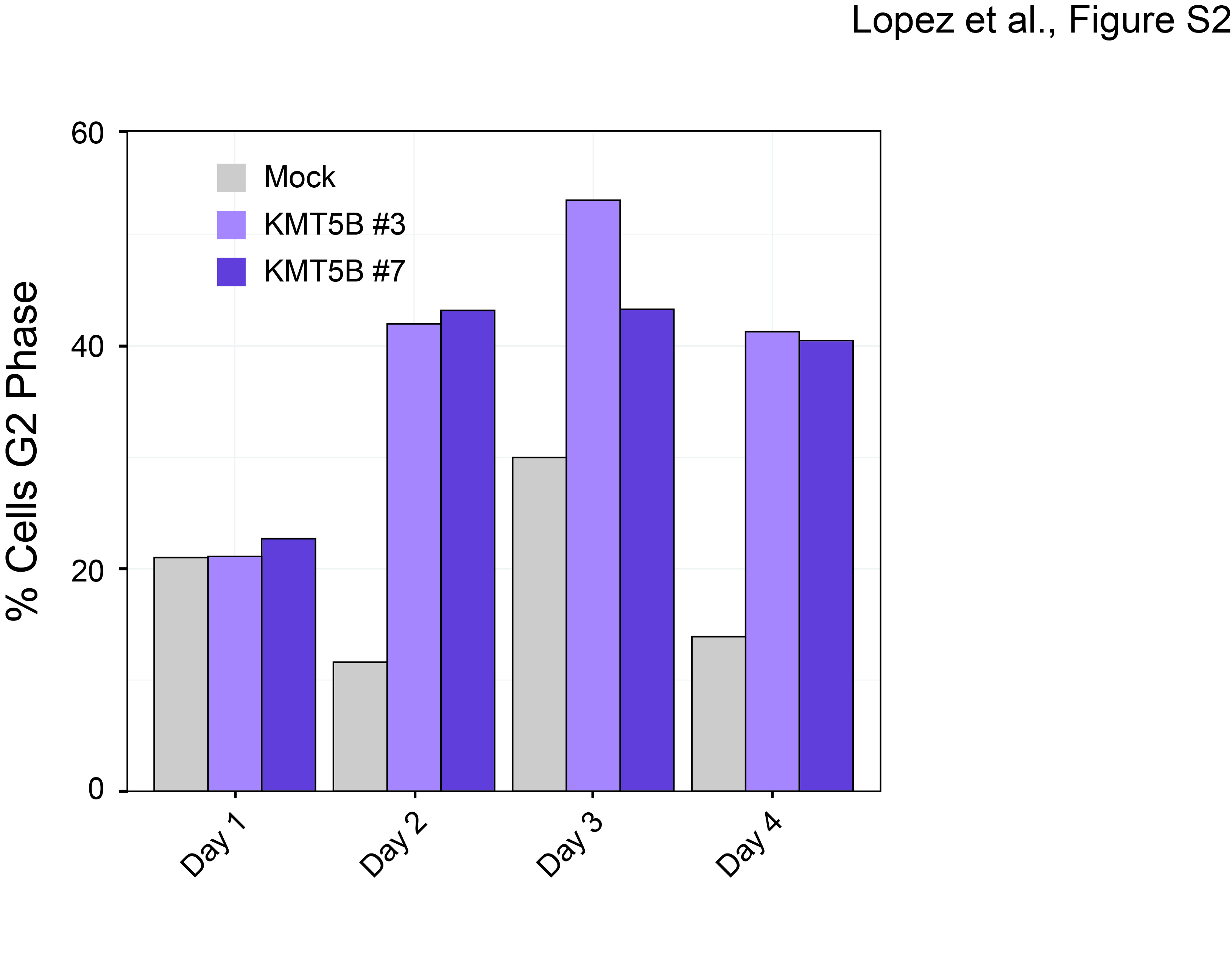

Supplement: Supplementary file 2 [file Image_2.JPEG]

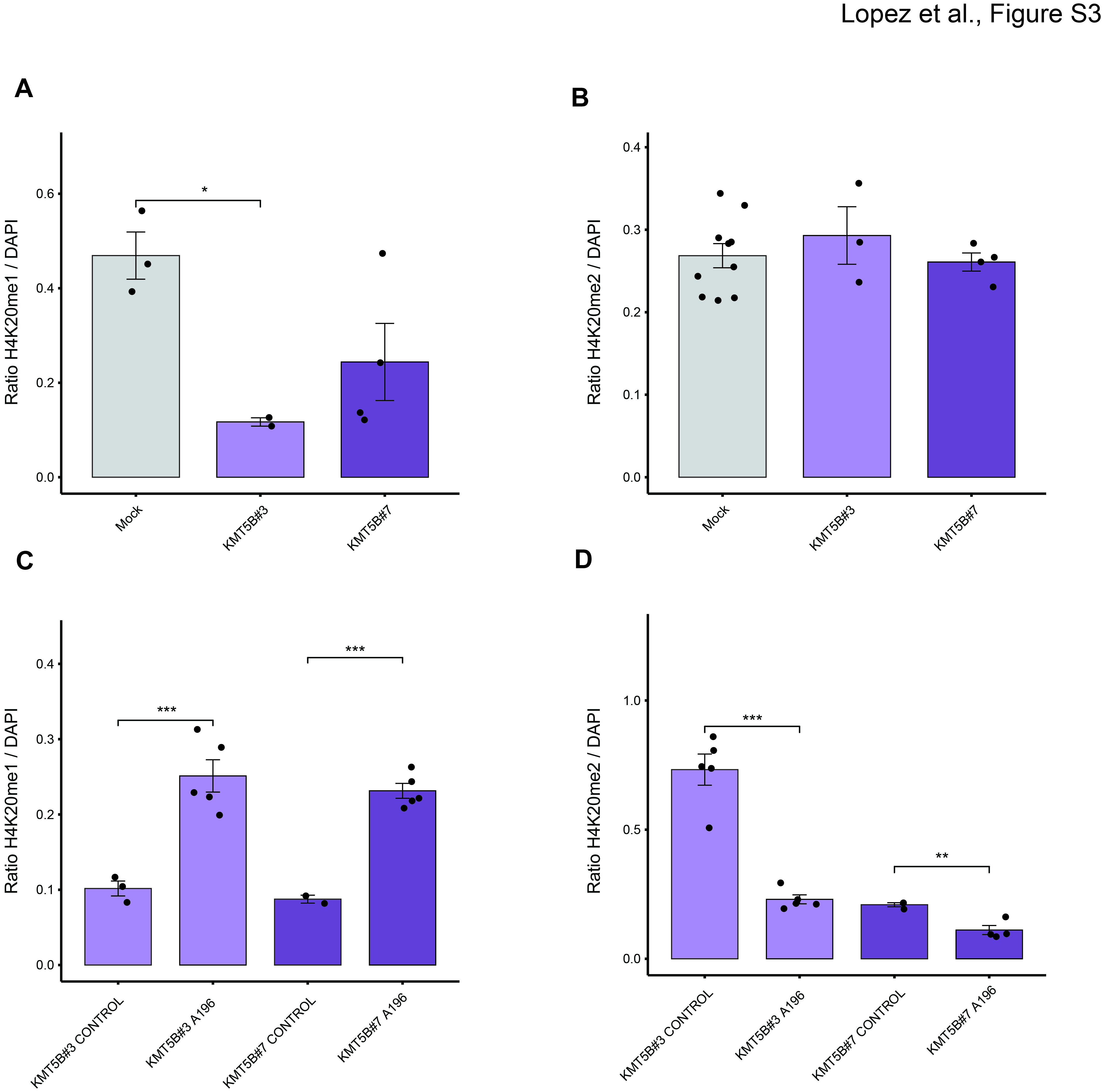

Supplement: Supplementary file 3 [file Image_3.JPEG]

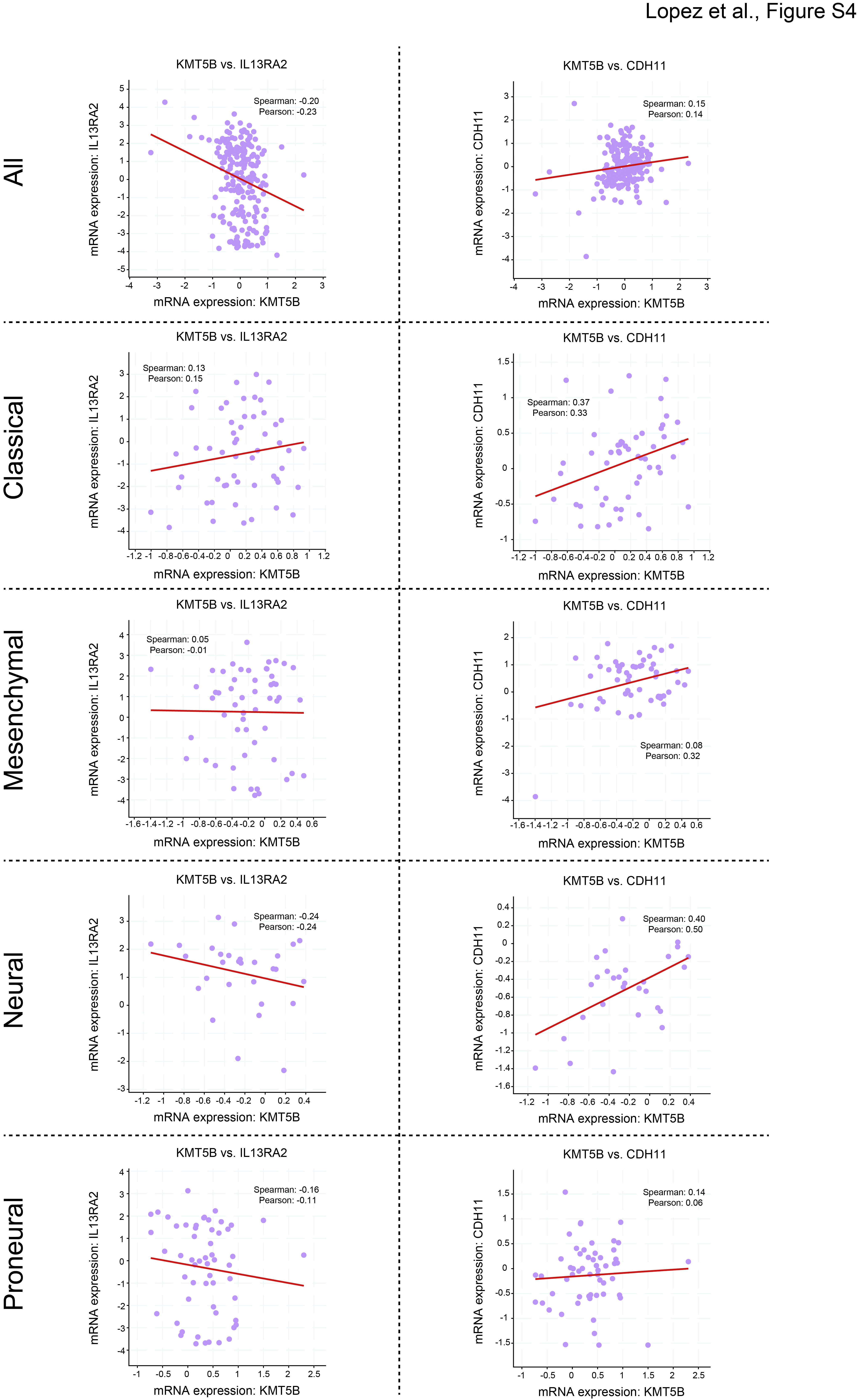

Supplement: Supplementary file 4 [file Image_4.JPEG]
