# Supplementary material for: Epigenetic Deregulation of the Histone Methyltransferase KMT5B Contributes to Malignant Transformation in Glioblastoma
Source: Front Cell Dev Biol. 2021 Aug 10;9:671838. doi: 10.3389/fcell.2021.671838 (PMC8383299; doi:10.3389/fcell.2021.671838)
Supplement: Supplementary file 13 [file Data_Sheet_1.PDF]

SUPPLEMENTARY MATERIAL

Lopez et al., 2021

This document includes the following supplementary material:

- **SUPPLEMENTARY METHODS**
- **SUPPLEMENTARY TABLES AND FIGURES**
- **SUPPLEMENTARY TABLE AND FIGURE LEGENDS**
- **SUPPLEMENTARY REFERENCES**

Supplementary Methods:

Gene Expression Arrays
Raw CEL files from normal brain, purified cell types (astrocytes, microglia), brain tumor samples and LN-229 cells were obtained from ArrayExpress under the accession numbers E-GEOD-36634, E-GEOD-15824and E-GEOD-23806 (Günther et al. 2008; Sim, Windrem, and Goldman 2009; Grzmil et al. 2011). Additional information regarding clinical or pathological status and accession numbers of these particular samples is provided in Supplementary **Table S8**. Microarray analyses were performed using the R/Bioconductor package affy (version 1.54.0). Raw data were normalized by the robust multi-array averaging method (RMA) and the intensity of the probe 222566_at was selected for the estimation of KMT5B levels. A matrix including normalized log2 expression values was used for representation purposes, and statistical significance was calculated by means of the Welch t-test method. Pairwise correlation analyses between KMT5B and candidate genes was performed using the cBioPortal platform (Cerami et al. 2012) with microarray data obtained from to the comprehensive characterization of glioblastoma performed by the TCGA consortium (Cancer Genome Atlas Research Network 2008).

Supplementary Table S1. List of primers used in the study.

Supplementary Table S2. List of antibodies used in the study.

Supplementary Table S3. 5mC and 5hmC array estimates for *KMT5B*.

Supplementary Table S4. Whole Genome Bisulfite Sequencing data for *KMT5B*.

Supplementary Table S5. Pyrosequencing results for *KMT5B*.

Supplementary Table S6. RNAseq statistics.

Supplementary Table S7. Gene abundance and DESeq2 statistics corresponding to RNAseq experiment.

Supplementary Table S8. Gene expression array data for KMT5B.

Supplementary Figure S1. Expression of KMT5B in non-tumoral brain, and normal brain cellular populations *versus* oligodendroglioma, astrocytoma, GBM and GBM cell line LN-229. Box plots represent the gene expression levels of *KMT5B* in different brain datasets obtained from Günther et al. and the cBio Cancer Genomics Portal (Günther et al. 2008; Cerami et al. 2012). Each dot corresponds to the expression level of a given sample. Statistical significance between the different subgroups is indicated (Welch t-tests; *$p < 0.05$, **$p < 0.01$, ***$p < 0.001$).

Supplementary Figure S2. Cell cycle analysis to confirm G2/M arrest induced by KMT5B overexpression. Control and KMT5B-expressing cells were stained with propidium iodide and analyzed by flow cytometry. The percentage of cells in G2 was determined using FlowJo software and plotted for control and KMT5B-expressing clones to confirm that overexpression of KMT5B causes cell cycle arrest in G2, as previously shown by Evertts et al. (Evertts et al. 2013).

Supplementary Figure S3. Quantification of the immunofluorescence analysis of mono- and dimethylation of H4K20 shown in figure 4. Fluorescence intensity measurements of DAPI and H4K20me1 (A, C) or H4K20me2 (B, D) in mock and KMT5B-transfected clones were performed using the ZEN lite software. Graphics represent relative intensity units for H4K20me1 or me2 normalized with respect to DAPI (Welch t-test; *$p < 0.05$, **$p < 0.01$, ***$p < 0.001$).

Supplementary Figure S4. Correlation analysis of KMT5B levels and expression of downstream candidate genes. Data was obtained from the TCGA consortium (Cancer Genome Atlas Research Network 2008) using the cBio Cancer Genomics Portal (Cerami et al. 2012). Scatter plots represent the correlation between KMT5B and IL13RA2 or CDH11 in all the samples from the GBM dataset (n= 206, top graph), or in those samples grouped by their corresponding GBM gene expression profile (Classical, Mesenchymal, Neural and Proneural). Spearman and Pearson's correlations for each comparison are indicated.

Supplementary References:

Cancer Genome Atlas Research Network. 2008. 'Comprehensive Genomic Characterization Defines Human Glioblastoma Genes and Core Pathways'. *Nature* 455 (7216): 1061–68. https://doi.org/10.1038/nature07385.

Cerami, Ethan, Jianjiong Gao, Ugur Dogrusoz, Benjamin E. Gross, Selcuk Onur Sumer, Bülent Arman Aksoy, Anders Jacobsen, et al. 2012. 'The CBio Cancer Genomics Portal: An Open Platform for Exploring Multidimensional Cancer Genomics Data'. *Cancer Discovery* 2 (5): 401–4. https://doi.org/10.1158/2159-8290.CD-12-0095.

Evertts, Adam G., Amity L. Manning, Xin Wang, Nicholas J. Dyson, Benjamin A. Garcia, and Hilary A. Coller. 2013. 'H4K20 Methylation Regulates Quiescence and

Chromatin Compaction'. *Molecular Biology of the Cell* 24 (19): 3025–37. https://doi.org/10.1091/mbc.E12-07-0529.

Grzmil, Michal, Pier Morin, Maria Maddalena Lino, Adrian Merlo, Stephan Frank, Yuhua Wang, Gerald Moncayo, and Brian A. Hemmings. 2011. 'MAP Kinase-Interacting Kinase 1 Regulates SMAD2-Dependent TGF-β Signaling Pathway in Human Glioblastoma'. *Cancer Research* 71 (6): 2392–2402. https://doi.org/10.1158/0008-5472.CAN-10-3112.

Günther, H. S., N. O. Schmidt, H. S. Phillips, D. Kemming, S. Kharbanda, R. Soriano, Z. Modrusan, H. Meissner, M. Westphal, and K. Lamszus. 2008. 'Glioblastoma-Derived Stem Cell-Enriched Cultures Form Distinct Subgroups According to Molecular and Phenotypic Criteria'. *Oncogene* 27 (20): 2897–2909. https://doi.org/10.1038/sj.onc.1210949.

Sim, Fraser J., Martha S. Windrem, and Steven A. Goldman. 2009. 'Fate Determination of Adult Human Glial Progenitor Cells'. *Neuron Glia Biology* 5 (3–4): 45–55. https://doi.org/10.1017/S1740925X09990317.
